# Re-evaluating herd protection by Vi typhoid vaccine in a cluster randomized trial

**DOI:** 10.1093/inthealth/ihz069

**Published:** 2019-10-14

**Authors:** Mohammad Ali, Dipika Sur, Suman Kanungo, Firdausi Qadri, Deok Ryun Kim, Taufiqul Islam, Justin Im, Faisal Ahmmed, Yun Chon, Ashraful Islam Khan, K Zaman, Florian Marks, Shanta Dutta, Sujit K Bhattacharya, John D Clemens

**Affiliations:** 1 Johns Hopkins Bloomberg School of Public Health, 615, N Wolfe Street, Baltimore, MD-21205, USA; 2 National Institute of Cholera and Enteric Diseases, P-33 CIT Road, Scheme XM, Beliaghata, Kolkata, India; 3 International Centre for Diarrhoeal Disease Research, Bangladesh, 68 Shaheed Tajuddin Ahmed Sarani Mohakhali, Dhaka 1212, Bangladesh; 4 International Vaccine Institute, SNU Research Park, 1 Gwanak-ro, Gwanak-gu, Seoul, Republic of Korea; 5 Department of Medicine, University of Cambridge, Cambridge, UK; 6 UCLA Fielding School of Public Health, Los Angeles, CA 90095-1772, USA

**Keywords:** cluster randomized trial, fried egg design, typhoid fever, vaccine effectiveness, Vi polysaccharide vaccine, Clinical trial registration: ClinicalTrials.gov, NCT00125008

## Abstract

**Background:**

In a cluster randomized trial (CRT) of a Vi polysaccharide vaccine against typhoid in the slums of Kolkata we found evidence of vaccine herd protection. However, transmission of typhoid into clusters from the outside likely occurred in this densely populated setting, which could have diminished our estimates of vaccine herd protection.

**Methods:**

Eighty clusters (40 in each arm) were randomised to receive a single dose of either Vi or inactivated hepatitis A vaccine. We analysed protection for the entire cluster and for subclusters consisting of residents of the innermost households.

**Results:**

During 2 y of follow-up, total protection was 61% (95% CI 41 to 75), overall protection was 57% (95% CI 37 to 71) and indirect protection was 44% (95% CI 2 to 69). Analyses of the innermost 75% and 50% of households of the clusters showed similar findings. However, in the innermost 25% of households of the clusters, total protection was 82% (95% CI 48 to 94) and overall protection was 66% (95% CI 27 to 84). There was not a sufficient sample size to demonstrate such a trend for indirect protection in these innermost households.

**Conclusions:**

The findings suggest that analyses of the entire cluster may have led to underestimation of herd protection against typhoid by Vi vaccine and that restriction of the analyses to the inner subclusters may have led to a more accurate estimation of vaccine herd effects.

## Introduction

Typhoid fever, caused by *Salmonella enterica* serovar Typhi (*S.* Typhi), remains an important cause of morbidity and mortality in populations residing in low- and middle-income countries (LMICs), with global estimates ranging between 11 and 21 million cases and 128 000 to 161 000 deaths annually.^1^^,^^2^ An injectable Vi polysaccharide vaccine is safe and moderately effective,^3,4^ and has been licensed for persons ≥2 y of age.^5^ However, its use in public health programmes has been limited despite a recommendation for its use by the WHP Strategic Advisory Group of Experts.^5^ Surveillance for typhoid in Guangxi, China after the introduction of Vi vaccine without massive structural change in the water sanitation and hygiene infrastructure for the general population revealed near disappearance of the disease,^6^ raising the possibility that the vaccine can elicit herd as well as direct protection.

To address whether Vi vaccine confers herd as well as direct protection against typhoid, we conducted a large-scale, cluster randomized trial (CRT) of Vi vaccine given to persons ≥2 y of age in Kolkata, India, where typhoid fever is endemic.^7^ Analysis of typhoid in the clusters revealed evidence of herd protection. However, because transmission of typhoid into the clusters from outside their perimeters likely occurred in this densely populated urban setting, and because such transmission would be predicted to depress estimates of vaccine herd protection, we decided to reanalyse the trial data using the ‘fried egg’ approach. This approach, in which only the innermost households of clusters (the ‘yolk’) are analysed, with the residents outside the innermost clusters (the ‘white’) serving as a buffer, has been claimed to minimize biased estimates of herd protection due to inward transmission.^8^ Herein we report the results of a reanalysis of the trial,^7^ which suggests that inward transmission of typhoid likely resulted in underestimates of Vi vaccine herd protection in our original published analysis.

## Methods

### Study site

The trial was carried out in a portion of Ward 29 and all of Ward 30 in eastern Kolkata, a legally registered urban slum with a population of about 62 000. A census of the *de jure* population was undertaken prior to vaccination to enumerate members of all households, including their geographical coordinates of residence, and to collect the socio-economic and water and sanitation status of each household. Each individual in the census was assigned a unique study identification number so that the individual could be followed over the period of the study. The census, together with household mapping, was used to define 80 contiguous geographical clusters that served as the units of randomization.

### Randomization of clusters

**Figure 1:**
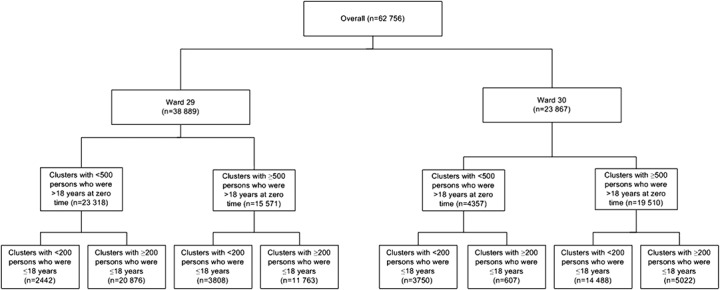
Diagrammatic presentation showing subjects’ distribution within strata and clusters.

We divided the clusters into eight strata, defined by study ward, number of residents who were ≤18 y of age at zero time (<200 vs ≥200 persons) and number of residents who were >18 y of age (<500 vs ≥500 persons) (Figure 1), and randomized the clusters in blocks of two within each stratum to create the Vi vaccine and hepatitis A vaccine (HAV) arms of the trial.

### Vaccination

Each dose of Vi vaccine (Typherix, GlaxoSmithKline) contained 25 μg of Vi polysaccharide. The control agent, inactivated HAV (Havrix, GlaxoSmithKline), contained 720 IU of inactivated hepatitis A virus for children 2–18 y of age and 1440 IU for adults. Each vaccine was administered by i.m. injection in a participant and observer blinded fashion. The vaccines were administered between 27 November and 31 December 2004. Residents ≥24 mo of age, without subjective or objective fever and not pregnant or lactating, were eligible to receive vaccine after giving written informed consent (guardian in case of minors). Vaccination was done in project vaccination centres set up in each cluster.

### Surveillance for enteric fever

Five project clinics (three in Ward 29 and two in Ward 30) were established to conduct surveillance for febrile illnesses and to refer patients with severe disease for their primary care. Private medical care providers in the two study wards were encouraged to refer their febrile patients to these study clinics. Additionally, the emergency rooms, outpatient clinics and inpatient wards of the two government hospitals serving the study area monitored patients presenting with febrile illnesses. Subjects from the study area presenting with a history of at least 3 d of fever were examined by a study physician, data on the subject’s history and physical findings were systematically recorded and a blood specimen was collected for culture after obtaining verbal informed consent. Standard biochemical and serological methods were used to identify *S.* Typhi.^9^ Patients were identified in the surveillance sites with the assistance of study census identification cards carried by patients and an onsite computerized database. Whenever a blood culture yielded *S.* Typhi, a study team was dispatched to the home of the patient within 7 d to verify that the subject whose name had been given had indeed visited the treatment site for care on the date noted in the surveillance. Patients received antibiotic treatment following the national guidelines.

### Definitions

Zero time, the onset of follow-up, was the date of vaccination or, for non-vaccinees, the median date of vaccination for vaccinees in the cluster. A febrile episode comprised all treatment visits for fever in which the recalled onset of fever for one visit was within 14 d of presentation for the next earlier visit. An episode of typhoid fever was a febrile episode in which *S.* Typhi was isolated from at least one blood culture during the febrile episode. The onset of the episode was the recalled onset of fever for the first treatment visit of the episode.

### Defining the yolk for the fried egg analytic approach

**Figure 2:**
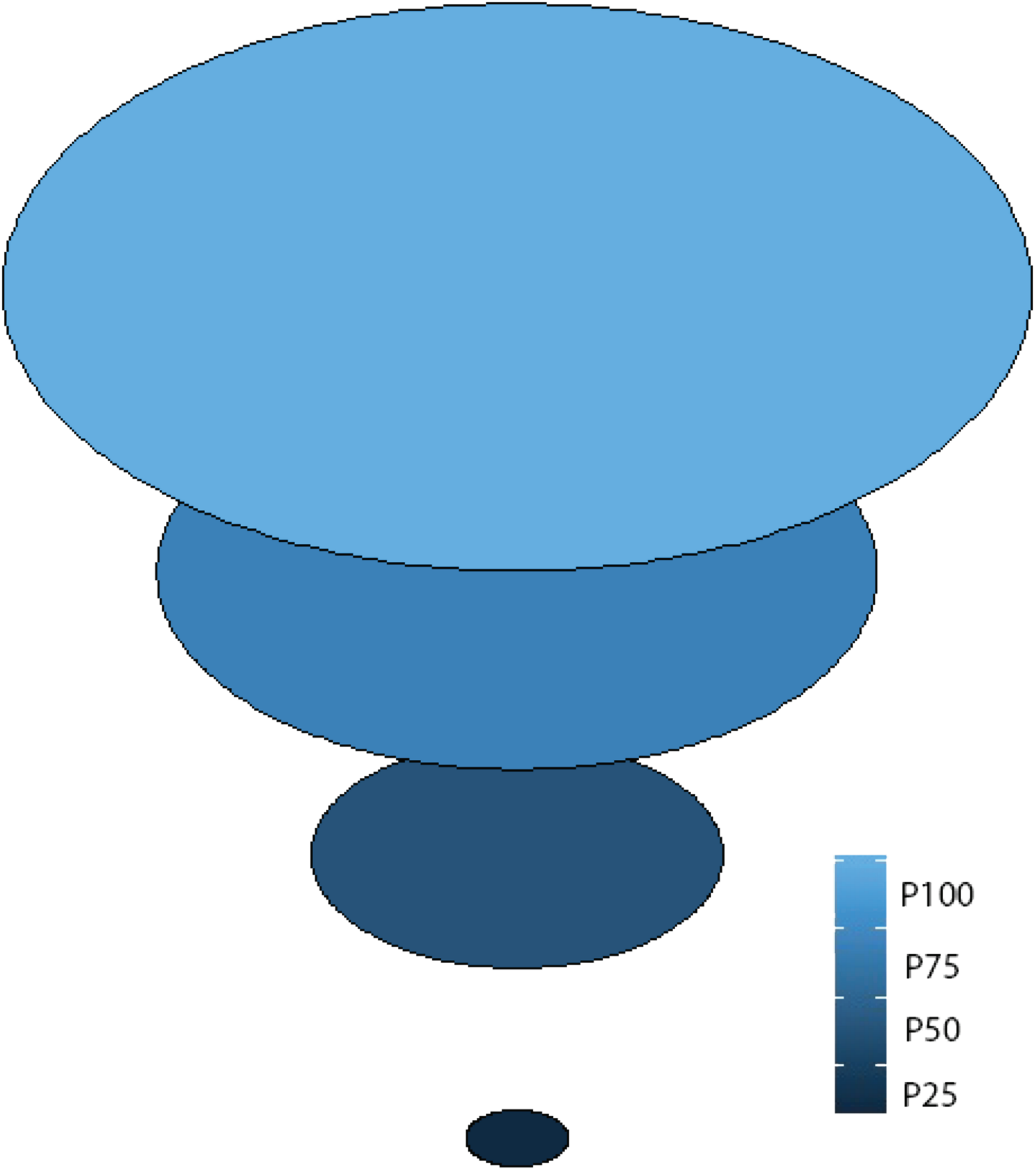
Diagrammatic representation of the four fractions of the fried egg approach.

**Figure 3:**
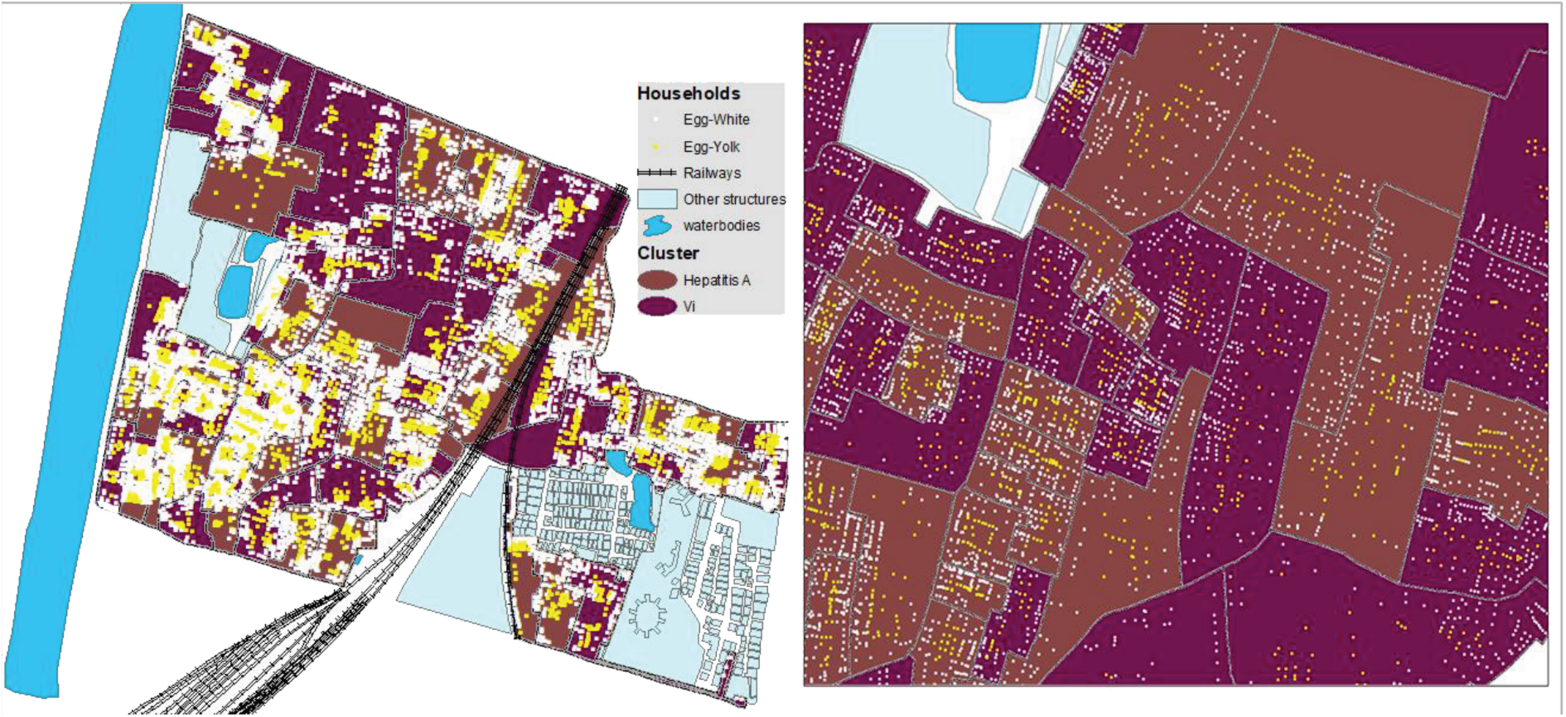
Distribution of study area households (entire study area in the left panel, close-up view of some of the clusters in the right panel) for analyses of the P25 clusters. Note: The yellow dots are the households in the P25 clusters and the white dots represent other households in the entire cluster.

We used the fried egg approach to reanalyse the data for this trial.^8^ With this approach we analysed Vi protection against typhoid fever for the entire cluster, as well as for residents of the innermost 75%, the innermost 50% and the innermost 25% of households (‘yolks’) of each cluster. A diagram of these four fractions is shown in Figure 2. We hypothesized that if estimates of Vi herd protection against typhoid had been attenuated by the transmission of typhoid fever into the clusters from the outside, this protection would be most evident in the innermost households. To demarcate these different sized yolks, we calculated the linear distance of each household to the nearest cluster perimeter and sorted the households in each cluster in ascending (furthest from to closest to the nearest perimeter) order by distance. We then assembled successive proportions of households, beginning with the household furthest from the perimeter and proceeding to include households progressively closer to the nearest perimeter until the desired fraction of households was reached. Before undertaking the analysis, we specified four fractions of households for analysis: 25% (innermost yolk), 50%, 75% and 100% (outermost yolk including the entire cluster), referred to as P25, P50, P75 and P100, respectively. Figure 3 shows households that were included in analyses of overall Vi protection for the P25 group.

### Analytic strategies

As in our original analysis of this trial, the occurrence of blood culture-proven typhoid fever constituted the primary outcome for the trial.^7^ All measures of Vi vaccine protection against culture-proven typhoid fever were expressed as the proportionate reduction of disease incidence ([1−hazard ratio of typhoid fever in the Vi clusters versus the HAV clusters]×100%). We assessed Vi vaccine herd protection as total, indirect and overall vaccine protection. For assessment of total protection, protection directly conferred to Vi vaccinees as well as additional herd protection of Vi vaccinees, we compared rates of typhoid in Vi vaccine recipients in the Vi clusters versus rates in HAV recipients in the HAV clusters. For indirect protection, protection of non-vaccinees against typhoid in the Vi clusters due to reduced person-to-person transmission of typhoid in the Vi clusters, we compared rates of typhoid among non-vaccinees in the Vi clusters versus rates among non-vaccinees in the HAV clusters. And for overall protection, protection of all members of the Vi-vaccinated clusters due to direct protection of Vi vaccinees and additional herd protection of Vi vaccinees and non-vaccinees, we compared rates of typhoid among all residents of the Vi clusters versus rates among all residents of the HAV clusters.^10^ If transmission of typhoid into the clusters from the outside had occurred, we hypothesized that estimates of Vi vaccine herd protection should become more pronounced the longer the distance of the household from the nearest perimeter. As in our earlier analysis,^7^ we limited our analyses to persons present at zero time and we analysed each of these measures of Vi protection after redefining the persons under analysis in the clusters as the P25, P50, P75 and P100 populations. In all analyses we evaluated Vi vaccine protection against the first episodes of typhoid fever having onsets between 1 and 730 d after zero time, and for which a home visit confirmed that the person whose name was given at the treatment centre had indeed sought care on the date of presentation.

We evaluated the following *a priori* zero time variables as potential confounding variables in analyses of vaccine protection: age (years), sex (male vs female), religion (Hindu vs others), ability of head of household to read and write (yes vs no), monthly per capita household expenditure (<median vs ≥median), ownership of at least one luxury item in household (yes vs no), household use of a tube well or faucet for drinking water (yes vs no), cluster population density (<median vs ≥median), flush toilet in household (yes vs no), access to a specific place for waste disposal in the household (yes vs no) and distance of household to the nearest treatment centre (<median vs ≥median). Comparisons of these variables between treatment arms employed generalized estimating equations to adjust for membership in the randomized clusters, with the logit link function for dichotomous variables and the identity link function for dimensional variables.^11^

To estimate vaccine protection, we conducted survival analyses, right-censoring individuals who died or migrated out before the end of the follow-up period or the end of the follow-up period, whichever came first. We fitted Cox proportional hazards regression models for each of the redefined yolks and each type of Vi vaccine herd protection.^12–14^ These models were adjusted for age at zero time, sex and the stratification variables for randomization, as well as variables found to be unequally distributed at p<0.05 in bivariate baseline comparisons of the arms of the study, following the requirement for at least 10 events per covariate to maximize the coverage of the CI of the estimate from the regression model.^15^ HRs were estimated by exponentiating the coefficient for the Vi vaccine variable in these models and vaccine protective effectiveness (PE) was estimated as ([1−HR]×100%). SEs for the coefficients were used to estimate p-values and 95% CIs for the HRs. All statistical analyses were performed using SAS version 9.4 (SAS Institute, Cary, NC, USA).

### Ethics

The institutional review boards of the International Vaccine Institute, the National Institute of Cholera and Enteric Diseases, India and the Indian Council of Medical Research approved the protocol and monitored the progress of the study. Individual written informed consent was obtained from all participants or their guardians. There were no agreements regarding the confidentiality of the data between study sponsors and the investigators.

## Results

The assembly of subjects and outcomes for analysis has been published previously.^7^ At zero time there were 62 756 residents in the 80 trial clusters, of whom 61 280 were age eligible for the trial. A total of 18 869 individuals received the Vi vaccine and 18 804 individuals received the HAV. The two treatment groups were well balanced at the individual level with respect to demographic, socio-economic and water source and hygiene characteristics (Tables S1–S12).

For analysis of overall Vi vaccine protection (protection of all residents in the Vi clusters relative to all residents in the HAV clusters) against typhoid fever, there were 62 756 individuals and 177 typhoid fever episodes in the P100 group (Table 1), 46 978 persons and 129 typhoid fever episodes in the P75 group, 31 216 persons and 87 episodes in the P50 group and 15 469 persons and 36 episodes in the P25 group (Table 1). Correspondingly, overall PE against typhoid fever was 57% (95% CI 37 to 71; p<0.0001) for the P100 group, with similar values for the P75 group (PE 51% [95% CI 26 to 67]; p=0.0005) and P50 group (PE 51% [95% CI 21 to 70]; p=0.0032). Overall protection rose in the P25 group to 66% (95% CI 27 to 84; p=0.005) (Table 1).

**Table 1:** Overall, total and indirect protection by Vi vaccine against typhoid fever in the differently defined clusters

Measures of protection	Vi vaccine clusters			HAV clusters					
	No. of persons	No. of cases	Rate/1000 person-years	No. of persons	No. of cases	Rate/1000 person-years	PE^a^ (%)	95% CI (%)	p-Value
P100 clusters									
Overall^a^	31 075	50	0.84	31 681	127	2.10	57	37 to 71	<0.0001
Total^b^	18 869	34	0.93	18 804	96	2.65	61	41 to 75	<0.0001
Indirect^c^	12 206	16	0.70	12 877	31	1.28	44	2 to 69	0.0429
P75 clusters									
Overall	23 148	40	0.90	23 830	89	1.96	51	26 to 67	0.0005
Total	14 079	26	0.96	13 935	66	2.46	58	34 to 74	0.0002
Indirect	9060	14	0.82	9895	23	1.24	33	−30 to 64	0.2502
P50 clusters									
Overall	15 566	27	0.91	15 650	60	2.01	51	21 to 70	0.0032
Total	9512	16	0.87	9210	46	2.59	62	32 to 78	0.0010
Indirect	6060	11	0.96	6440	14	1.16	14	−81 to 59	0.6897
P25 clusters									
Overall	7767	9	0.60	7702	27	1.86	66	27 to 84	0.0054
Total	4914	4	0.42	4413	22	2.61	82	48 to 94	0.0016
Indirect	2840	5	0.93	3283	5	0.82	−14	−298 to 68	0.8427

^a^Overall protection for the P100, P75 and P50 clusters was adjusted for the variables used to stratify the clusters for randomization, age, religion, living in a household with a monthly per capita expenditure above the median and living in a household with a longer distance to the nearest treatment centre than the median. For the P25 clusters, it was adjusted only for the variables used to stratify the clusters for randomization.

^b^Total protection for the P100 and P75 clusters was adjusted for the variables used to stratify the clusters for randomization, age, religion, living in a household with a monthly per capita expenditure above the median and living in a household with a specific place for waste disposal. For the P50 clusters it was adjusted for the variables used to stratify the clusters for randomization, age, religion and living in a household with a monthly per capita expenditure above the median. For the P25 clusters it was adjusted only for the variables used to stratify the clusters for randomization.

^c^Indirect protection for the P100 clusters was adjusted for the variables used to stratify the clusters for randomization, age and living in a household with a longer distance to the nearest treatment centre. For the P75 clusters it was adjusted for the variables used to stratify the clusters for randomization and age. For the P50 clusters it was adjusted only for the variables used to stratify the clusters for randomization. For the P25 clusters, no variables were used to adjust the effectiveness.

For evaluation of total protection (protection of Vi recipients relative to HAV recipients), there were 37 673 individuals and 130 cases of typhoid fever in the P100 group, 28 014 individuals and 92 episodes of typhoid fever in the P75 group, 18 722 individuals and 62 episodes of typhoid fever in the P50 group and 9327 individuals and 26 episodes of typhoid fever in the P25 group (Table 1). Estimates of total Vi vaccine protection against typhoid fever were 61% (95% CI 41 to 75; p<0.0001) in the P100 group, with similar estimates for the P75 (PE 58% [95% CI 34 to 74]; p=0.0002) and P50 (PE 62% [95% CI 32 to 78]; p=0.001) groups. Again, protection rose in the P25 group (82% [95% CI 48 to 94]; p=0.0016).

In analyses of indirect vaccine protection (protection of non-recipients of vaccine in the Vi clusters relative to non-recipients of vaccine in the HAV clusters), there were 25 083 individuals and 47 episodes of typhoid fever in the P100 group, 18 955 individuals and 37 episodes of typhoid fever in the P75 group, 12 500 individuals and 25 episodes of typhoid fever in the P50 group and 6123 individuals and 10 episodes of typhoid fever in the P25 group (Table 1). Indirect protection against typhoid fever was 44% (95% CI 2 to 69; p=0.0429) in the P100 group, 33% (95% CI −30 to 64; p=0.25) in the P75 group (Table 1), 14% (95% CI −81 to 59; p=0.69) in the P50 group and −14% (95% CI −298 to 68; p=0.8427) in the P25 group (Table 1).

## Discussion

Although the conventional analysis of this CRT, in which entire clusters were analysed, revealed clear evidence of herd protection by Vi vaccine, we believe that the estimates might have been biased in a conservative direction due to likely transmission of typhoid into the clusters in the densely populated urban setting for the trial, as observed in a trial conducted in Bangladesh using the oral cholera vaccine.^16^ When we attempted to remove the effects of such transmission on estimates of Vi herd protection by restricting the analysis to the innermost population of the clusters (P25 group), we found a much higher level of total Vi protection (82%) and a somewhat higher level of overall Vi protection (66%) than observed in the analysis of the entire cluster. While the point estimate of indirect Vi protection in the central 25% of households of the clusters did not reveal a similar trend, the wide confidence limits surrounding the estimate (95% CI −298 to 68%) did not exclude an increase and were limited by a very small number of typhoid outcomes for this analysis. However, it is not only that the indirect protection did not show the expected increase due to a small number of outcome events in the P25 group, but also the CI for total protection for the P100 and P25 groups overlapped. Since the P25 group was a subset of the P100 group, there is no real statistical basis for comparing the two groups. In aggregate, our findings are consistent with our hypothesis that transmission of typhoid into the clusters attenuated our original estimates of Vi herd protection and that restriction of the analysis to the innermost households acted to produce estimates of protection that were less affected by this bias.

To a certain extent our study might seem somewhat anachronistic, as the fact that Vi polysaccharide vaccine is no longer considered an attractive vaccine for control of typhoid fever in endemic settings is attested to by Gavi’s decision not to support the vaccine in its typhoid vaccine introduction programme. Recently the WHO has prequalified for purchase by United Nations agencies a Vi polysaccharide–tetanus toxoid conjugate typhoid vaccine (Vi-TT) that is licensed in India. Compared with Vi polysaccharide, this Vi conjugate vaccine is more immunogenic and induces immunological memory.^17^ And Gavi has opened a funding window that subsidizes LMICs meeting Gavi criteria to acquire this vaccine for their public health programmes. Nonetheless, our analysis has important implications for clinical evaluations of this and other future Vi-conjugate vaccines, for which CRTs will be needed to estimate their population-level effectiveness, including vaccine herd protection, when deployed in realistic public health programmes.

To provide evidence for policy deliberations about deployment of the WHO-prequalified Vi-TT in urban slums of South Asian cities, where typhoid often thrives, we are currently testing this vaccine in a large cluster randomized effectiveness trial in the urban slums of Dhaka, Bangladesh.^18^ Although the CRT is considered the best design for measuring population-level vaccine herd protection in an unbiased fashion,^10^ a caveat of this assertion is that clusters are selected and defined in such a fashion that they represent self-contained epidemiological units of transmission of typhoid. In practice, including at the field site for our ongoing CRT of Vi-TT in Dhaka, it will be uncommon for selected clusters to meet this condition, and estimates of vaccine herd protective effects may be diminished to the extent that this assumption is violated.^19^ Buffer zones are commonly used in CRTs to prevent transmission of the target pathogen and diffusion of interventions between clusters. However, if typhoid is transmitted from the surrounding buffer zones into the study clusters, buffer zones will not necessarily safeguard against this problem. Because it may be difficult in advance to predict whether such transmission will occur in a CRT of vaccines, a conservative approach would be to use the fried egg analytic approach to help safeguard against this bias, and our current trial of Vi-TT is designed to anticipate the use of this analytic approach.

The major limitation of using the fried egg approach is the sample size required to achieve adequate power in the analysis. Our analysis of the innermost population (P25) clearly had an inadequate sample size and hence inadequate statistical power to measure the indirect protection provided by Vi vaccine in this setting. Thus use of the fried egg approach to analyse vaccine herd effects in CRTs requires consideration of the balance between the increased cost associated with the larger sample sizes of the clusters and the increased accuracy of estimation of vaccine herd effects in the innermost clusters.

In conclusion, our reanalysis of the CRT of Vi polysaccharide in Kolkata using the fried egg approach yielded evidence of higher levels of certain types of Vi vaccine herd protection in the innermost populations of the randomized clusters compared with our original analyses of entire clusters. These findings sound a note of caution that CRTs of vaccines may underestimate vaccine herd protection when epidemiological assumptions about transmission of the target pathogen are not fulfilled and underscore the need to consider using the fried egg analytic approach at the design stage of such CRTs.
